# Perioperative nursing management of an adult patient with giant right-sided Bochdalek hernia: a case report

**DOI:** 10.3389/fmed.2026.1809018

**Published:** 2026-03-10

**Authors:** Xiaohui Yang, Yunping Sun, Yuhan Li, Jinxin Chen, Zhihui Li, Tingting Li, Meng Peng, Sha Li, Ai Huang

**Affiliations:** 1 Operating Room, Union Hospital, Tongji Medical College, Huazhong University of Science and Technology, Wuhan, China; 2 Intensive Care Unit, Union Hospital, Tongji Medical College, Huazhong University of Science and Technology, Wuhan, China; 3 Department of Hepatobiliary Surgery, Union Hospital, Tongji Medical College, Huazhong University of Science and Technology, Wuhan, China; 4 Department of Thoracic Surgery, Union Hospital, Tongji Medical College, Huazhong University of Science and Technology, Wuhan, China

**Keywords:** adult diaphragmatic hernia, Bochdalek hernia, enteral nutrition, herniorrhaphy, perioperative nursing, pulmonary rehabilitation, self-efficacy

## Abstract

**Background:**

Adult right-sided Bochdalek hernia is extremely rare and poses unique perioperative nursing challenges, especially with intrathoracic displacement of abdominal organs.

**Case presentation:**

A 19-year-old male presented with exertional dyspnea. Imaging revealed a giant right-sided diaphragmatic hernia containing part of the liver, gallbladder, and colon, with approximately 70% compression of the right lung.

**Interventions:**

A multidisciplinary team developed individualized perioperative plans. Prehabilitation based on self-efficacy theory was implemented, including respiratory training, nutritional support. During surgery, a surgical risk early-warning and rapid response system was established. Precise intraoperative pressure injury risk assessment and dynamic positioning management were implemented. Continuous dynamic temperature monitoring and individualized target temperature management were applied to prevent perioperative hypothermia. Postoperative care focused on airway management,sequential respiratory support, early gastrointestinal function monitoring, and progressive pulmonary rehabilitation.

**Results:**

The patient was discharged after 32 days without major complications. Follow-up at 3, 6, and 12 months showed full recovery and no recurrence.

**Conclusion:**

Comprehensive, individualized perioperative nursing interventions can optimize recovery and outcomes in complex adult right-sided diaphragmatic hernia repair.

## 1 Introduction

Bochdalek hernia is a rare congenital diaphragmatic defect resulting from incomplete diaphragm development and failure of closure of the posterolateral pleuroperitoneal folds, leading to a posterolateral diaphragmatic defect. Approximately 80–85% of cases occur on the left side, 10–15% on the right, and bilateral hernias are exceedingly rare (<2%) (1). In neonates, the condition often presents with severe respiratory distress, cyanosis, tachypnea, and abdominal flattening due to lung compression by herniated abdominal organs (2). In adults, Bochdalek hernia may cause mild dyspnea, chest pain, abdominal distension, or nausea, but is frequently asymptomatic and detected incidentally on imaging (3). While neonatal cases are more common, occurring in approximately 1 in 2,500 live births and predominantly left-sided, adult right-sided Bochdalek hernia is extremely rare, with an incidence of only 0.17% (4). Fewer than 200 adult cases have been reported in the English-language literature to date (5). Guidelines recommend early surgical repair once diagnosed, even in asymptomatic patients, to prevent potentially life-threatening complications (6, 7). Reports on perioperative nursing for adult right-sided giant Bochdalek hernia are scarce, and relevant experience remains limited. On July 8, 2024, our thoracic surgery department admitted an adult patient with a giant right-sided Bochdalek hernia, containing the liver, gallbladder, and colon, who presented with exertional dyspnea. Through multidisciplinary team (MDT) collaboration and precise perioperative nursing, the patient recovered successfully and was discharged after 32 days. One-year follow-up showed recovery. The following reports our nursing experience.

## 2 Case presentation

### 2.1 General information

**Figure 1:**
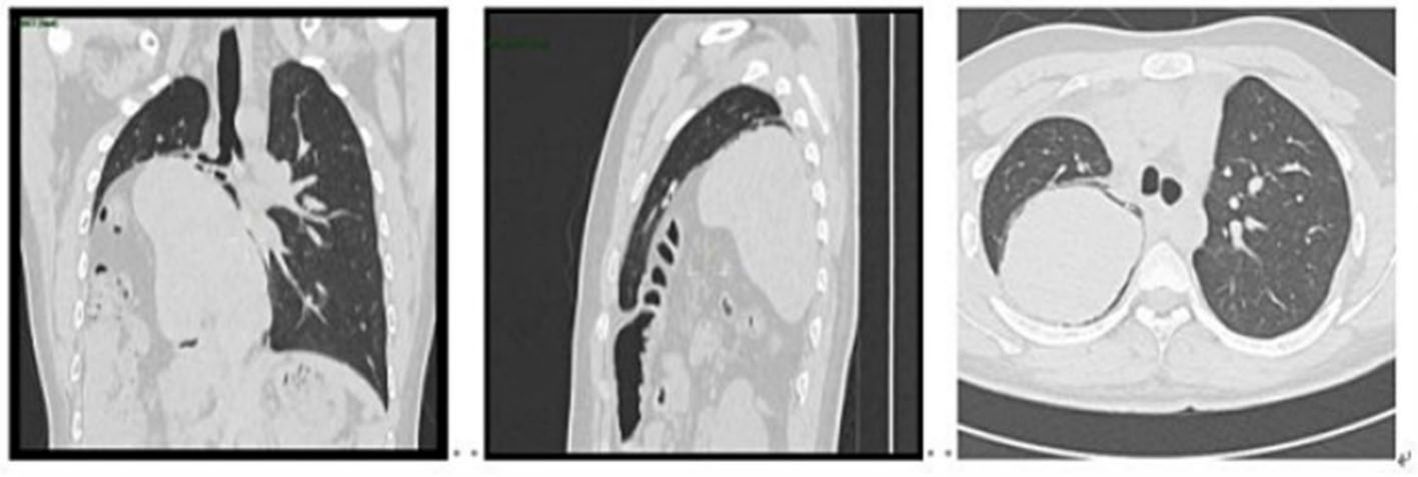
Preoperative chest CT showing right-sided diaphragmatic hernia with intrathoracic displacement of the liver and mediastinal shift.

A 19-year-old male, an unmarried student, previously healthy, presented with exertional dyspnea that began three months prior during physical activity. He had no history of cardiovascular or respiratory disease and denied hypertension, diabetes, chronic or infectious diseases, prior trauma, smoking, regular medication use, family history of hereditary disorders, or food and drug allergies. On July 8, 2024, he was admitted to the thoracic surgery department, where further evaluation led to a diagnosis of diaphragmatic hernia. Physical examination revealed clear consciousness, temperature 36.3 °C, heart rate 109 bpm, respiratory rate 20/min, blood pressure 112/87 mmHg, oxygen saturation 90%, and BMI 25.86. Chest CT demonstrated a right-sided diaphragmatic hernia with partial liver herniation, lower-lobe lung compression (approximately 70%), and mediastinal shift (as shown in Figure 1). Upper gastrointestinal contrast study revealed duodenal bulb filling defects, irregular mucosa, and proximal jejunal malposition with abnormal rotation. Pulmonary function testing indicated severe mixed ventilatory impairment (FVC 45.4% predicted, FEV1/VC 73.75% predicted). The patient was diagnosed with non-traumatic right-sided Bochdalek hernia, with herniation of the liver, gallbladder, and colon, right-lung compression causing atelectasis, mediastinal shift, and severe mixed ventilatory dysfunction.

### 2.2 Treatment process and outcomes

**Figure 2:**
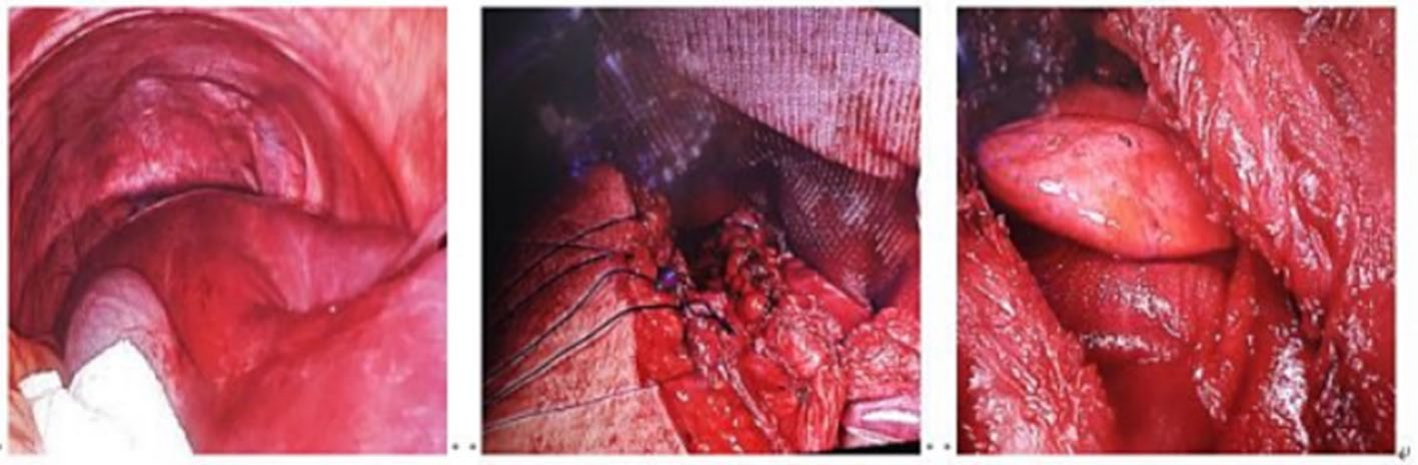
Intraoperative view of thoracoabdominal diaphragmatic hernia repair with mesh reinforcement.

**Figure 3:**
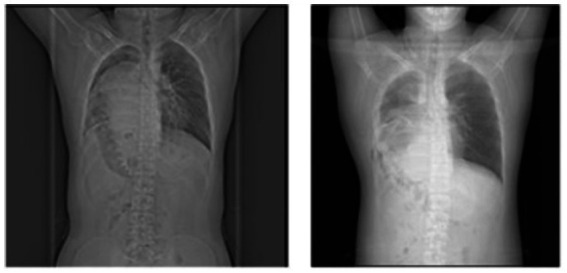
Postoperative chest X-ray showing lung re-expansion.

The patient underwent comprehensive preoperative evaluation and, on July 17, 2024, underwent laparoscopic-assisted right thoracoabdominal diaphragmatic hernia repair under general anesthesia with double-lumen endotracheal intubation and one-lung ventilation. The procedure included complex thoracoabdominal adhesiolysis and diaphragmatic defect repair with mesh reinforcement. After standard skin preparation, a combined thoracoabdominal approach was performed under laparoscopic guidance. Intraoperative exploration included the diaphragm, liver, and portions of the colon and small intestine. The displaced internal organs were gradually repositioned back into the abdominal cavity. The diaphragmatic defect was closed with non-absorbable sutures, and a 15 cm × 20 cm biological mesh was placed on the thoracic side for reinforcement(as shown in Figure 2). Postoperatively, the patient was transferred to a negative-pressure single-room ICU, receiving sedation, analgesia, anti-infective therapy, fluid resuscitation, and mechanical ventilation. On postoperative day (POD) 2, Laboratory tests showed elevated inflammatory markers (CRP 105.23 mg/L, WBC 16.43 × 10^9/L, neutrophils 13.51 × 10^9/L). Chest CT revealed right lung atelectasis with severe infection, prompting fiberoptic bronchoscopy and alveolar lavage. On POD 4, tracheostomy for airway optimization. On POD 5, marked abdominal distension was noted; bedside ultrasonography indicated gastric content retention, consistent with delayed gastric emptying. Enteral nutrition support was initiated. On POD 9, the patient was conscious with stable vital signs, the patient was transferred to the thoracic surgery ward for continued rehabilitation, with a tracheal cannula and jejunal feeding tube in place. Smoothly discharged after 32 days of hospitalization. Follow-up at 1 year showed no recurrence. Preoperative and postoperative chest X-ray imaging, (as shown in Figure 3.). To enhance clarity and transparency of the perioperative course, a standardized chronological timeline including diagnostic findings, therapeutic decisions, nursing interventions, and outcome indicators is provided in Table 1.

**Table 1:** Standardized timeline of diagnosis, treatment, nursing interventions, and outcomes.

Time point	Key findings	Clinical decisions and treatment	Nursing interventions	Outcome indicators
2024-07-08 (Admission)	Dyspnea after 3 months of physical activity; pulmonary function: extremely severe mixed ventilatory dysfunction	Admitted to thoracic surgery; diagnosis: non-traumatic right-sided diaphragmatic hernia	Low-flow oxygen to maintain SpO₂ Bed rest to reduce oxygen consumption Admission education	SpO₂ 90% (moderate hypoxemia); patient and family understood condition
2024-07-12	CT: right diaphragmatic hernia, partial liver herniation, mediastinal shift	MDT consultation; complete preoperative evaluation; optimize respiratory function	Preoperative pulmonary rehabilitation	Self-efficacy improved from low to high
2024-07-17 (Surgery day)	Intraoperative: diaphragmatic defect, thoracoabdominal adhesions, herniated abdominal viscera	Laparoscopic-assisted right thoracoabdominal diaphragmatic hernia repair; adhesiolysis; mesh reinforcement	Intraoperative coordination: surgical risk early-warning system Pressure injury prevention Positioning and temperature management Transfer to single-room ICU postoperatively	Surgery completed successfully
Post-op 24 h (POD 1)	Acute postoperative phase; delayed emergence; poor respiratory function	ICU admission; sedation, analgesia, anti-infective therapy; mechanical ventilation	Ventilator management Airway humidification and suctioning Vital-sign monitoring Pain and sedation assessment	Mechanical ventilation support ongoing
POD 2	Elevated inflammatory markers: CRP 105.23 mg/L, WBC 16.43 × 10^9^/L, NEU 13.51 × 10^9^/L; CT: right lung atelectasis, severe infection	Fiberoptic bronchoscopy with bronchoalveolar lavage	Pre-procedure preparation and intraoperative cooperation Enhanced airway drainage Chest physiotherapy Monitor temperature, respiratory status, sputum	Pulmonary infection confirmed; treated with lavage
POD 4	Persistent impaired ventilation; excessive airway secretions	Tracheostomy to optimize airway management	Prepare tracheostomy equipment Post-tracheostomy airway care, fixation, humidification	Airway management optimized; no ventilator-associated pneumonia
POD 5	Abdominal distension; bedside ultrasound: gastric retention	Diagnose delayed gastric emptying; initiate enteral nutrition	Gastrointestinal decompression Neostigmine 1 mg IM Glycerin enema Enteral feeding tube care Monitor abdominal distension and gastric residual	Enteral nutrition successfully initiated
POD 9	Conscious, stable vital signs	Transfer from ICU to thoracic surgery ward with tracheal cannula, jejunal feeding tube, and central venous catheter	Continuous cardiac monitoring Respiratory rehabilitation Tracheostomy care Nutritional support Early mobilization guidance	Clinical condition stabilized; transferred to general ward
POD 32 (Discharge)	Good recovery; no dyspnea; independent ambulation	Discharge after rehabilitation	Discharge education: respiratory training, activity guidance, nutrition, follow-up plan	Discharged uneventfully
1-Year Follow-Up	No discomfort; no recurrence	Scheduled follow-up	Long-term respiratory function maintenance education	No recurrence; favorable prognosis

## 3 Discussion

### 3.1 Preoperative nursing care

#### 3.1.1 Multidisciplinary team establishment and surgical-nursing planning

This patient presented with a rare adult congenital giant right-sided Bochdalek hernia with intrathoracic visceral displacement. Surgical success and postoperative recovery directly impact long-term quality of life. To optimize outcomes, a multidisciplinary team—including thoracic surgery, hepatobiliary surgery, anesthesiology, ICU, nutrition, operating room, and rehabilitation were established preoperatively. Comprehensive discussions and nursing risk assessments addressed potential challenges such as inadequate preparation, massive intraoperative bleeding, cardiac arrest, delayed gastrointestinal recovery, and postoperative pulmonary rehabilitation needs. An optimal surgical plan, targeted nursing interventions, and contingency strategies were developed to ensure coordinated perioperative management.

#### 3.1.2 Preoperative prehabilitation nursing based on self-efficacy theory

Self-efficacy refers to an individual’s confidence in successfully completing a specific task, directly influencing behavioral motivation (8, 9). Preoperative prehabilitation can enhance physical function and nutritional status, enabling patients to undergo surgery in optimal condition, reduce postoperative complications, and accelerate recovery (10). Based on self-efficacy theory, preoperative prehabilitation was guided by the responsible nurse. Self-efficacy assessment: The Chinese version of the General Self-Efficacy Scale [revised by Wang et al. was used (11)], with a pre-prehabilitation score of 19, indicating low self-efficacy. (1) Video-based education: One-on-one pulmonary rehabilitation training was provided in combination with video instruction. Each morning at 9:00 a.m., a 5-min hospital-produced video (including diaphragmatic breathing and respiratory trainer demonstrations) was played at the bedside. After the video, the responsible nurse answered questions individually and corrected the patient’s technique. Studies (12, 13) have shown that abdominal breathing combined with a respiratory training device can strengthen respiratory muscle function, improve coordination of respiratory muscle groups, increase lung ventilation, and promote gas exchange efficiency. A respiratory function training device is an active inspiratory training tool that improves pulmonary function, enhances respiratory muscle strength, and reduces atelectasis, and it has been widely used in clinical pulmonary rehabilitation (13). (2) Respiratory Trainer Combined with Diaphragmatic Breathing. The charge nurse instructed the patient to perform diaphragmatic breathing training combined with the use of a respiratory training device. The training protocol was as follows. Firstly, hand hygiene was performed. Secondly, resistance was set to level 3; the patient assumed a sitting position at the edge of the bed and inhaled deeply through the nose for 1 s to approximately 80% of lung capacity. Thirdly, the patient sealed the mouth around the mouthpiece, inhaled for 1 s, held the breath for 2 s, and then exhaled slowly over 3 s. Fourth, 10–20 breathing cycles were repeated, followed by 2–3 huff coughing maneuvers. For diaphragmatic breathing, the patient was placed in a relaxed position, inhaled deeply, then opened the mouth to produce a “ha” sound while contracting the abdominal muscles, and finally coughed with both hands supporting the abdomen. This sequence constituted one training set. Daily respiratory training using the respiratory trainer was performed in three sets, with a two-hour interval between sets. Each session lasted 15–20 min, and the prehabilitation program continued for seven consecutive days. After completion of the seven-day program, self-efficacy was reassessed using the General Self-Efficacy Scale, yielding a score of 35, which indicated high self-efficacy.

### 3.2 Intraoperative nursing

#### 3.2.1 Establish a surgical risk early-warning and rapid emergency response system

This patient presented with a rare adult giant right-sided Bochdalek hernia, making the surgery complex and high-risk. Beyond routine operative support, the circulating nurse focused on potential intraoperative emergencies, particularly injuries to the liver, major vessels, or heart that could result in massive hemorrhage or cardiac arrest. A surgical risk early-warning and rapid response system was established,with the plan detailed as follows: Ensuring Effective Circulatory Volume: Preoperatively, an 18G peripheral line was established. Simultaneously, assistance was provided to the anesthesiologist in performing central venous and arterial catheterization. Autologous blood salvage equipment was prepared, and an individualized blood management plan was developed. During surgery, the cell-saver system collected blood from operative bleeding and hemostatic gauze, gently irrigated with heparinized saline to further increase the total volume of salvageable blood. Establishment of a Goal-Oriented Precision Fluid Management System: Hemodynamic parameters were used to guide the development of fluid management plans. Central venous pressure, mean arterial pressure, urine output, lactate, and other key indicators were dynamically monitored to accurately assess the patient’s circulating volume status and guide fluid resuscitation. Blood products were administered based on coagulation test results, and electrolyte and acid–base imbalances were promptly corrected according to blood gas analysis. Intraoperatively, the patient received 4,500 mL of crystalloid solution and 2,000 mL of colloid solution, with a urine output of 1,000 mL and blood loss of 1,300 mL. Additionally, 280 mL of allogeneic washed red blood cells, 200 mL of fresh frozen plasma, and 660 mL of autologous blood salvage were transfused. The patient’s vital signs remained stable throughout the procedure, with no transfusion-related adverse events, and circulatory status was maintained. Preoperative Emergency Equipment Preparation: One day prior to surgery, the circulating nurse prepared emergency equipment in the operating room according to the contingency plan, including a defibrillator, crash cart, bedside ultrasound machine, three sets of suction devices, thoracotomy and laparotomy emergency instruments, various vascular sutures, and absorbable sutures. Adequate supply and readiness of these items ensured rapid response to any urgent situation.

#### 3.2.2 Precise intraoperative pressure injury risk assessment and dynamic positioning

(1) Pressure Injury Risk Assessment: To prevent acquired intraoperative pressure injury (AIPI), the circulating nurse conducted a comprehensive preoperative assessment using the 2018 version of the 3S Pressure Injury Risk Assessment Scale, independently developed by our operating room team (14). The preoperative score was 20, indicating moderate risk. The intraoperative dynamic score averaged 14.44, indicating high risk. (2) Positioning Management: Dynamic strategies were implemented to optimize surgical exposure and efficiency. Based on operative requirements, the patient was placed in a modified supine position with 15° head-up tilt and 30° right-side elevation, utilizing gravity to expose the right diaphragmatic defect and reduce resistance during liver repositioning. During positioning, nurses followed the principles of symmetry, support, and pressure relief, using gel pads (5 cm thick, medium firmness) and soft pillows (5–8 cm high) to ensure even pressure distribution at all support points, maintaining pressure below 32 mmHg. During mesh fixation, the surgical table was adjusted to a horizontal position to ensure balanced suture tension and facilitate diaphragmatic reconstruction. (3) Nursing Interventions: Preoperatively, a 20 cm-diameter gel head support was placed, 10 × 10 cm disposable foam dressings were applied to the scapula and sacrococcygeal region, and both heels were elevated 30–40° using soft pillows (5–7 cm above the bed surface). Gaps between the body and surgical table including the neck, waist, and popliteal fossa, were filled with 2–3 cm thick non-linting medical cotton pads to maintain functional positioning and prevent muscle stretch or localized pressure. (4) Evidence suggests that hourly “micro-movements” reduce intraoperative pressure injury incidence (15). Considering surgical exposure and sterile requirements, we implemented a modified “micro-movement” protocol:every 2 h, the surgical table level function was activated to tilt the patient 15° alternately left or right for 5 min, preventing sustained local pressure. Care was taken to avoid additional compression from surgical instruments, energy devices, or staff activities. (5) Postoperatively, the patient’s skin condition was handed over to the ICU team, with particular attention to the color, temperature, and integrity of pressure-prone areas. With these measures, no intraoperative pressure injuries occurred in this patient.

#### 3.2.3 Continuous dynamic temperature monitoring and individualized targeted temperature management

In this case, the patient had a large surgical incision, prolonged exposure of body cavities, and a surgery duration exceeding 10 h. substantial fluid and blood transfusions, and anesthesia, all of which are high-risk factors for intraoperative hypothermia. Studies indicate that pre-warming the operating room for over 25 min before anesthesia can raise skin temperature and prevent redistribution hypothermia (16). Therefore, a dynamic, stage-based perioperative temperature management strategy was implemented to maintain core temperature above 36 °C (17). The nursing interventions were as follows (1) Dynamic warming measures: One hour before surgery, the operating table and bedding were preheated using an inflatable warming device, and the room temperature was set to 24 °C. During surgery, the room was maintained at 22–24 °C, the warming device set at 40 °C, and all intravenous fluids, blood products, and irrigation solutions were warmed before administration. Postoperatively, until the patient left the operating room, room temperature was maintained at 24 °C to prevent further heat loss. (2) Real-Time Temperature Monitoring: Following anesthesia induction, an 8 cm reusable nasopharyngeal temperature probe was placed for continuous core temperature monitoring. Alarm thresholds were set at ≤36 °C to enable timely adjustments to warming interventions, achieving precise, dynamic temperature control. With this strategy, the patient’s intraoperative core temperature fluctuated between 36.0 and 37.0 °C, and no hypothermia occurred.

### 3.3 Postoperative care

#### 3.3.1 Refined airway management for pulmonary complication prevention

Studies indicates that early extubation reduces the incidence of ventilator-associated pneumonia (VAP), shortens ICU stay, and lowers hospitalization costs (18). However, chest CT on postoperative day (POD) 2 revealed bilateral inflammatory lesions with segmental atelectasis, underscoring the critical importance of airway management in this patient (19). (1) Airway clearance and humidification. Airway clearance techniques were implemented to control pulmonary infection. According to domestic expert consensus (19) and the UK guidelines for tracheal intubation management in critically ill patients (20), continuous normothermic and humidified airway support enhances secretion clearance (21). Airway temperature was maintained at 37 °C with 100% relative humidity; sterile water for Injection was replaced regularly and condensate promptly drained. The head of the bed was elevated to 30–45° within 6 h postoperatively, and cuff pressure was maintained at 25–30 cmH₂O. (2) Pharmacological therapy. Tan reqing injection (40 mL) diluted in 0.9% saline (100 mL) was administered intravenously once daily. From POD 3, nebulized beclomethasone dipropionate (0.8 g) combined with acetylcysteine (300 mg) was added three times daily. From POD 7, vibration-assisted sputum clearance was performed twice daily (10–15 Hz, 10–15 min/session twice daily), followed immediately by assisted expectoration. (3) Fiberoptic bronchoscopy combined with bronchoalveolar lavage. According to the American Association for Respiratory Care’s Clinical Practice Guidelines for Endotracheal Suctioning, bedside fiberoptic bronchoscopy may be used when secretions are excessive (22). On POD 2, bedside fiberoptic bronchoscopy with bronchoalveolar lavage was performed under appropriate sedation and analgesia. Sputum specimens were collected correctly, with daily assessment of sputum volume, color, and consistency, and close monitoring of culture results. (4) Infection control and basic care. Strict hand hygiene and aseptic techniques were enforced. Daily clothing changes, oral care with chlorhexidine solution, bed bathing, and eye care were provided three times daily. (5) Rational Use of Antimicrobial Agents. Routine monitoring of complete blood count, blood cultures, urine cultures, and sputum cultures was performed at intervals. Antimicrobial agents were selected in strict accordance with medical orders based on culture results and clinical findings, and adverse drug reactions were closely observed and documented. Cefoperazone–sulbactam (3.0 g) diluted in 100 mL of 0.9% sodium chloride was administered intravenously every 8 h. On postoperative day 3, chest CT revealed significant right lung infection; therefore, antimicrobial therapy was escalated with the addition of meropenem (1.0 g) diluted in 100 mL of 0.9% sodium chloride administered intravenously every 12 h. On postoperative day 7, sputum culture showed no bacterial growth. Following these nursing and therapeutic interventions, the patient was successfully weaned from mechanical ventilation on postoperative day 7, without developing ventilator-associated pneumonia or other severe complications.

#### 3.3.2 Sequential oxygen therapy for successful weaning

Preoperative CT imaging revealed partial herniation of the liver into the thoracic cavity, causing approximately 70% compression of the right lung. This markedly impaired the patient’s cough and sputum clearance, prolonged the need for mechanical ventilation, and increased the risk of pulmonary infection. To minimize ventilator-associated lung injury and promote pulmonary recovery, an individualized sequential oxygen therapy strategy was implemented postoperatively. (1) Initially, mechanical ventilation was provided using volume-controlled synchronized intermittent mandatory ventilation (V-SIMV) with the following parameters: FiO₂ 40%, tidal volume 420 mL, respiratory rate 16 breaths/min, and PEEP 8 cmH₂O (1 cmH₂O = 0.098 kPa). Arterial blood gas analysis 6 h after ICU admission showed oxygenation index 520 mmHg. Blood gases were monitored every 6–8 h, and ventilator parameters were dynamically adjusted to correct hypoxemia and optimize microcirculation. (2) From postoperative days (POD) 1–3, ventilation was maintained in pressure-controlled mode, with PEEP gradually titrated from 8 to 5 cmH₂O. Oxygenation index at the start of POD 1–3 was 417 mmHg. (3) On POD 4, due to delayed recovery of consciousness and persistent right lung compression (~35%), spontaneous breathing trials resulted in tachypnea (28 breaths/min) and tachycardia (120 bpm), indicating weaning failure. To reduce infection risk, bedside ultrasound-guided temporary tracheostomy was performed after informed consent. Simultaneously,music therapy was administered twice daily (30 min per session), using the patient’s preferred rap music played to alleviate stress. Post-tracheostomy, ventilatory support was transitioned to V-A/C mode (PEEP 5 cmH₂O, f 20 breaths/min, FiO₂ 30%), with oxygenation index ~560 mmHg. (4) On POD 5, ventilation was downgraded to continuous positive airway pressure (CPAP) with PEEP 5 cmH₂O, respiratory rate 25 breaths/min, and FiO₂ 28%, achieving oxygenation index ~553 mmHg. (5) On POD 6, a protocolized weaning strategy was initiated (23). After a successful spontaneous breathing trial, high-flow oxygen therapy was initiated. Nurses selected a well-fitting mask based on the patient’s level of cooperation and facial contours, and hydrocolloid dressings were applied to pressure points to prevent skin injury. During the weaning process, the patient’s needs were promptly assessed and addressed whenever possible, with positive reinforcement provided to enhance self-efficacy. Two hours later, respiratory rate increased to 28 breaths/min, heart rate 115 bpm, and fatigue was reported; mechanical ventilation was temporarily reinstated. Oxygenation index was ~478 mmHg. (6) On POD 7, the weaning protocol was reattempted, with successful transition to high-flow oxygen therapy (flow 60 L/min, FiO₂ 30%). Vital signs remained stable, oxygenation index reached 478 mmHg, and the patient reported no discomfort. (7) By POD 9, high-flow oxygen therapy continued at 50 L/min, FiO₂ 30%, with oxygenation index 533 mmHg, and was transferred out of the ICU. Overall, this individualized sequential respiratory support strategy effectively corrected hypoxemia and enabled a smooth transition to spontaneous breathing.

#### 3.3.3 Gastrointestinal function–guided individualized nutritional support

In patients with congenital giant right-sided diaphragmatic hernia, surgical repair is often associated with high repair tension and a rapid increase in intra-abdominal pressure after visceral reduction. When combined with perioperative immunosuppression and potential vagal nerve injury, postoperative gastrointestinal dysfunction is common. Therefore, close monitoring of abdominal signs was performed, including abdominal distension, pain, muscle tension, bowel sounds, abdominal circumference, and passage of flatus and stool, to promptly identify intestinal obstruction or hernia recurrence. (1) On postoperative day (POD) 2, physical examination revealed a soft, non-distended abdomen. Bowel sounds were 3 times/min, abdominal circumference was 89 cm, and no flatus or defecation was observed. According to the 2023 European Society for Clinical Nutrition and Metabolism (ESPEN) guidelines, Recommendation 7 is supported by Grade A evidence and carries a strong recommendation (24), enteral nutrition (EN) was initiated within 24–48 h postoperatively. Given a high aspiration risk score of 7, GRV monitoring serves as an objective indicator of feeding tolerance (25). Therefore, GRV was assessed in the right lateral position under ultrasound guidance by a certified ICU nurse. With the head of bed elevated 30–40°, GRV was measured once every 1–2 feedings using a convex probe to identify the gastric antrum cross-sectional area, which was converted to GRV using validated reference tables. (2) From POD 2–3, a total nutrition formula (500 mL) was administered via nasogastric tube at 30 mL/h. GRV ranged from 200-400 mL, with a feeding tolerance score of 1–2. (3)On POD 4, GRV increased to 400 mL with marked abdominal distension (tolerance score 3). Enteral nutrition was suspended. Gastrointestinal decompression was initiated (550 mL drainage), and intramuscular neostigmine (1 mg, q12h) and glycerin enema were administered. (4) On postoperative day 5, the patient developed marked abdominal distension. Abdominal circumference increased to 91 cm, and gastrointestinal decompression volume reached 950 mL. Bedside ultrasonography demonstrated gastric content retention, and a diagnosis of delayed gastric emptying was established. Gastrointestinal decompression was initiated, together with intramuscular neostigmine (1 mg) and glycerin enema. An endoscopic jejunal feeding tube was placed, and early enteral nutrition support was commenced. Enteral nutrition suspension (Peptison) was initiated at a total volume of 500 mL, delivered via the jejunal feeding tube at an infusion rate of 20 mL/h. (5) On postoperative day 8, no abdominal distension or other abnormalities were observed, and the gastrointestinal tolerance score was 0. Abdominal circumference decreased to 88 cm, and bowel sounds increased to 4 times/min. Enema administration and intramuscular neostigmine were discontinued. The volume of enteral nutrition suspension (Peptison) was increased from 500 mL to 1,000 mL. On postoperative day 9, at the time of transfer. On the day of transfer to the thoracic surgery ward. Thirst score (VAS 30) was recorded after transfer. Enteral nutrition suspension (Peptison) 500 mL was administered at an infusion rate of 40 mL/h. On postoperative day 10, enteral nutrition suspension (Peptison) 500 mL combined with potassium chloride injection (15 mL) was administered at an infusion rate of 30 mL/h. (6) Oral intake was initiated with semi-liquid foods on POD 15. With a protective, stepwise enteral nutrition strategy guided by dynamic gastrointestinal monitoring, gastrointestinal tolerance improved from a peak score of 3 to 0, allowing a smooth transition to full oral feeding without major complications such as aspiration pneumonia, gastrointestinal bleeding, or intestinal obstruction.

#### 3.3.4 Individualized progressive pulmonary rehabilitation

Early rehabilitation significantly improves exercise tolerance, reduces postoperative complications, enhances cardiopulmonary function, and improves prognosis (26, 27). In ICU patients, early passive and active rehabilitation has also been shown to shorten hospital stay, optimize resource utilization, and improve clinical outcomes (28, 29). In this patient, congenital right-sided giant diaphragmatic hernia led to right lung atelectasis and poor exercise tolerance, posing challenges to early pulmonary rehabilitation. According to the national expert consensus from Australia and New Zealand (30), following multidisciplinary consultation, an individualized pulmonary rehabilitation program was developed based on the patient’s clinical condition, level of consciousness, and degree of cooperation (Table 2).

**Table 2:** Individualized pulmonary rehabilitation training program.

Assessment Period.	Consciousness status	Rehabilitation protocol
Jul 18–20 (POD 1–3)	Sedated, mechanically ventilated	(1) Repositioned every 4 h; performed passive in-bed exercises to prevent muscle atrophy and deep vein thrombosis (DVT). (2) Head of bed elevated to 30–45°. (3) Passive range of motion (ROM) for all limbs; distal-to-proximal muscle massage, 3–5 min per limb, 3 times/day. (4) Elbow flexion/extension: 10–15 repetitions; knee flexion/extension: 10–15 repetitions; ankle 360° rotations: 10–15 repetitions.
Jul 21–23 (POD 4–6)	Conscious, intubated	(1) Bedside sitting: head of bed elevated to 90°, maintained 3 min, gradually increased to 5–15 min, 3 times/day. (2) Modified abdominal breathing: semi-recumbent, 8–10 breaths/min, 10 min per session, twice/day. (3) Intermittent pneumatic compression to prevent DVT.
July 24–25 (POD7-8d)	After removal of the endotracheal tube, a tracheostomy cannula was left in place	(1) Exercise training: The patient was instructed to perform in-bed pedal cycling exercises, with each set lasting 5 min, for a total of 10 min per session, twice daily. No fatigue was reported during training. Bedside mobility training was gradually intensified. The patient was encouraged to sit up at the bedside for 1 min, followed by dangling both legs for 1 min, then progressing to standing at the bedside for 1 min. The duration was incrementally increased to 5 min, without evident discomfort. (2) Respiratory training: The patient was guided to perform respiratory function training using a breathing exerciser, with three training sets conducted daily. During bedside mobilization and functional exercises, patient safety was ensured, and all indwelling tubes and lines were properly secured. Early out-of-bed mobilization was emphasized, as it facilitates attenuation of muscle atrophy and improves exercise tolerance and muscle strength.
Jul 26–Aug 5 (POD 9–19)	Conscious, tracheostomy, transferred to ward	(1) Exercise training: The patient was instructed to perform active ankle pump exercises, 20–30 repetitions per set, 3–4 sets per day. Quadriceps strengthening exercises were also performed, with 10 repetitions per set, 10 sets per session, 3–4 times daily. Assistance was provided for in-place marching exercises twice daily, each session lasting 5–10 min. (2) Respiratory training: Assisted the patient with chest percussion to facilitate sputum clearance and instructed family members to do the same. The patient was encouraged to cough independently to promote expectoration
Aug 6–9 (POD 20–23)	Conscious, tracheostomy removed and site sutured	(1) Pursed-lip breathing: Nasal inspiration with a 3–5 s breath-hold, followed by oral exhalation lasting 4–6 s, corresponding to an inspiration-to-expiration ratio of 1:2. Each session lasted 15 min and was performed twice daily. (2) Walking training: gradually increased distance from bedside to ward corridor; total ≥30 min/day, at least 3 days/week. (3) Achieved independent ward ambulation and good functional recovery at discharge.

## 4 Conclusion

Adult congenital right-sided giant diaphragmatic hernia is an exceptionally rare condition, and its nursing management poses substantial clinical challenges. Through systematic analysis and reflection on this case, we distilled several practice-informed insights: the establishment of a multidisciplinary collaborative model, the application of comprehensive risk assessment tools, the implementation of prehabilitation-oriented nursing strategies, and the delivery of individualized postoperative pulmonary rehabilitation. Collectively, these approaches may serve as a practical reference for the nursing management of similarly rare and complex conditions, particularly in enhancing care efficiency and optimizing recovery quality. Nevertheless, we became acutely aware of the inherent uncertainties encountered throughout the nursing process. The potential for abrupt clinical deterioration and pronounced interindividual variability necessitates sustained vigilance and dynamic adjustment of care plans to address emerging risks in a timely manner. Moreover, the intrinsic limitations of single-case evidence must be acknowledged. Given substantial patient heterogeneity and the absence of comparative data, the nursing strategies derived from this case cannot be directly generalized to all patients with comparable conditions.

## Data Availability

The original contributions presented in the study are included in the article/supplementary material, further inquiries can be directed to the corresponding author/s.
